# Heteropentanuclear Oxalato-Bridged *n*d–4f (*n*=4, 5) Metal Complexes with NO Ligand: Synthesis, Crystal Structures, Aqueous Stability and Antiproliferative Activity

**DOI:** 10.1002/chem.201502026

**Published:** 2015-08-10

**Authors:** Paul-Steffen Kuhn, Laura Cremer, Anatolie Gavriluta, Katarina K Jovanović, Lana Filipović, Alfred A Hummer, Gabriel E Büchel, Biljana P Dojčinović, Samuel M Meier, Annette Rompel, Siniša Radulović, Jean Bernard Tommasino, Dominique Luneau, Vladimir B Arion

**Affiliations:** a University of Vienna, Faculty of Chemistry, Institute of Inorganic Chemistry Währinger Strasse 42, 1090 Vienna (Austria); b Université Claude Bernard Lyon 1, Laboratoire des Multimatériaux et Interfaces (UMR 5615) Campus de la Doua, 69622 Villeurbanne cedex (France); c Institute for Oncology and Radiology of Serbia Pasterova 14, 11000 Belgrade (Serbia); d Universität Wien, Fakultät für Chemie, Institut für Biophysikalische Chemie Althanstraße 14, 1090 Wien (Austria); e Faculty of Chemistry, Institute of Analytical Chemistry, University of Vienna Währinger Strasse 38, 1090 Vienna (Austria); f University of Belgrade, Institute of Chemistry, Technology and Metallurgy, Center of Chemistry Studentski trg 12-16, Belgrade (Serbia); g Present address: Division for Physical Sciences and Engineering and KAUST Catalysis Center, King Abdullah University of Science and Technology Thuwal (Saudi Arabia)

**Keywords:** antitumor agents, lanthanide, nitrosyl, osmium, ruthenium

## Abstract

A series of heteropentanuclear oxalate-bridged Ru(NO)-Ln (4d–4f) metal complexes of the general formula (*n*Bu_4_N)_5_[Ln{RuCl_3_(μ-ox)(NO)}_4_], where Ln=Y (**2**), Gd (**3**), Tb (**4**), Dy (**5**) and ox=oxalate anion, were obtained by treatment of (*n*Bu_4_N)_2_[RuCl_3_(ox)(NO)] (**1**) with the respective lanthanide salt in 4:1 molar ratio. The compounds were characterized by elemental analysis, IR spectroscopy, electrospray ionization (ESI) mass spectrometry, while **1**, **2**, and **5** were in addition analyzed by X-ray crystallography, **1** by Ru K-edge XAS and **1** and **2** by ^13^C NMR spectroscopy. X-ray diffraction showed that in **2** and **5** four complex anions [RuCl_3_(ox)(NO)]^2−^ are coordinated to Y^III^ and Dy^III^, respectively, with formation of [Ln{RuCl_3_(μ-ox)(NO)}_4_]^5−^ (Ln=Y, Dy). While Y^III^ is eight-coordinate in **2**, Dy^III^ is nine-coordinate in **5**, with an additional coordination of an EtOH molecule. The negative charge is counterbalanced by five *n*Bu_4_N^+^ ions present in the crystal structure. The stability of complexes **2** and **5** in aqueous medium was monitored by UV/Vis spectroscopy. The antiproliferative activity of ruthenium-lanthanide complexes **2**–**5** were assayed in two human cancer cell lines (HeLa and A549) and in a noncancerous cell line (MRC-5) and compared with those obtained for the previously reported Os(NO)-Ln (5d–4f) analogues (*n*Bu_4_N)_5_[Ln{OsCl_3_(ox)(NO)}_4_] (Ln=Y (**6**), Gd (**7**), Tb (**8**), Dy (**9**)). Complexes **2**–**5** were found to be slightly more active than **1** in inhibiting the proliferation of HeLa and A549 cells, and significantly more cytotoxic than 5d–4f metal complexes **6**–**9** in terms of IC_50_ values. The highest antiproliferative activity with IC_50_ values of 20.0 and 22.4 μM was found for **4** in HeLa and A549 cell lines, respectively. These cytotoxicity results are in accord with the presented ICP-MS data, indicating five- to eightfold greater accumulation of ruthenium versus osmium in human A549 cancer cells.

## Introduction

Quite recently we became interested in ruthenium and osmium nitrosyl complexes with the prospect to create prodrugs able to release clinically effective levels of NO and metal complex within cancer cells.^1^–^3^ This took into account the fact that several classes of, mostly mononuclear, ruthenium and osmium coordination compounds have demonstrated promising anticancer potential both in vitro and in vivo.^4^ Moreover, the role of nitric oxide in several biological processes is well established and depends on its concentration in the cells.^5^, ^6^ It is beneficial at low level (<μM) but it may cause cell apoptosis at higher concentration of NO.^7^ In addition, ruthenium nitrosyl complexes are known for their electron-transfer properties and/or catalytic activity in organic synthesis, which are mainly based on the non-innocent character of the nitrosyl (NO) ligand.^8^ These complexes may also photo-release NO.^9^ Recently, we synthesized ruthenium and osmium nitrosyl complexes with azole heterocycles that were shown to undergo the *cis*–*trans* isomerization through a dissociative mechanism.^3^ We also evidenced much lower antiproliferative activity of the osmium complexes, in stark contrast to previous studies, where either smaller^10^, ^11^ or similar cytotoxicity^12^, ^13^ has been observed for related ruthenium and osmium complexes. Further, we investigated the effect of incorporating oxygen donors in the coordination sphere of the metal–nitrosyl complexes, starting with a series of osmium complexes with amino acids.^2^

Theranostic agents combining a targeted therapeutic drug and a diagnostic unit that fit the dose requirement for both the therapy and imaging would be ideally suited for cancer treatment allowing monitoring the therapy and response to therapy at the cellular level.^14^ Several imaging techniques are well used clinically for cancer diagnosis now, such as magnetic resonance imaging (MRI), single photon emission computed tomography (SPECT) or positron emission tomography (PET).^15^ Optical fluorescence imaging possesses a number of advantages such as high sensitivity, availability, excellent spatial and very fast temporal resolution allowing visualization and monitoring of the tumor cell biology in real time.^16^, ^17^ For in vivo purposes the use of MRI or PET is preferred to overcome problems of background fluorescence and photobleaching typical for fluorescence imaging, and, high absorption (e.g., hemoglobin) in the mid-visible range.^18^ Exploitation of photophysical properties of lanthanides (luminescence), and, in particular, of terbium and europium, characterized by long-lived (milliseconds timescale) excited states, is another way to avoid concerns related to fluorescence imaging. The long lifetimes provide an increase of a signal-to-noise ratio, since time-resolved fluorescence spectroscopy and microscopy can be used.

With this in mind, we turned our attention to lanthanide-labeling of ruthenium and osmium complexes with biologically active organic ligands. The use of luminescence emission from the lanthanide complexes in combination with ruthenium-based anticancer therapeutic drugs is indeed appealing for the design and synthesis of new potential cancer theranostics. At the early stage of development lanthanide labeling may allow the monitoring of subcellular distribution of potential drugs. In addition, lanthanide(III) salts themselves were found to exhibit moderate antiproliferative activity in vitro^19^, ^20^ as well as in vivo.^21^ These properties are related to their similarity to calcium ions, whereby lanthanide ions are higher charged and therefore show a strong affinity towards biological calcium binding sites.^19^, ^21^, ^22^ The anticancer activity could be further enhanced by complexation of lanthanide ions with various chelating and macrocyclic ligands such as chrysin (**A**),^23^ texaphyrins (**B**)^24^ or phenantroline derivatives (**C**).^25^
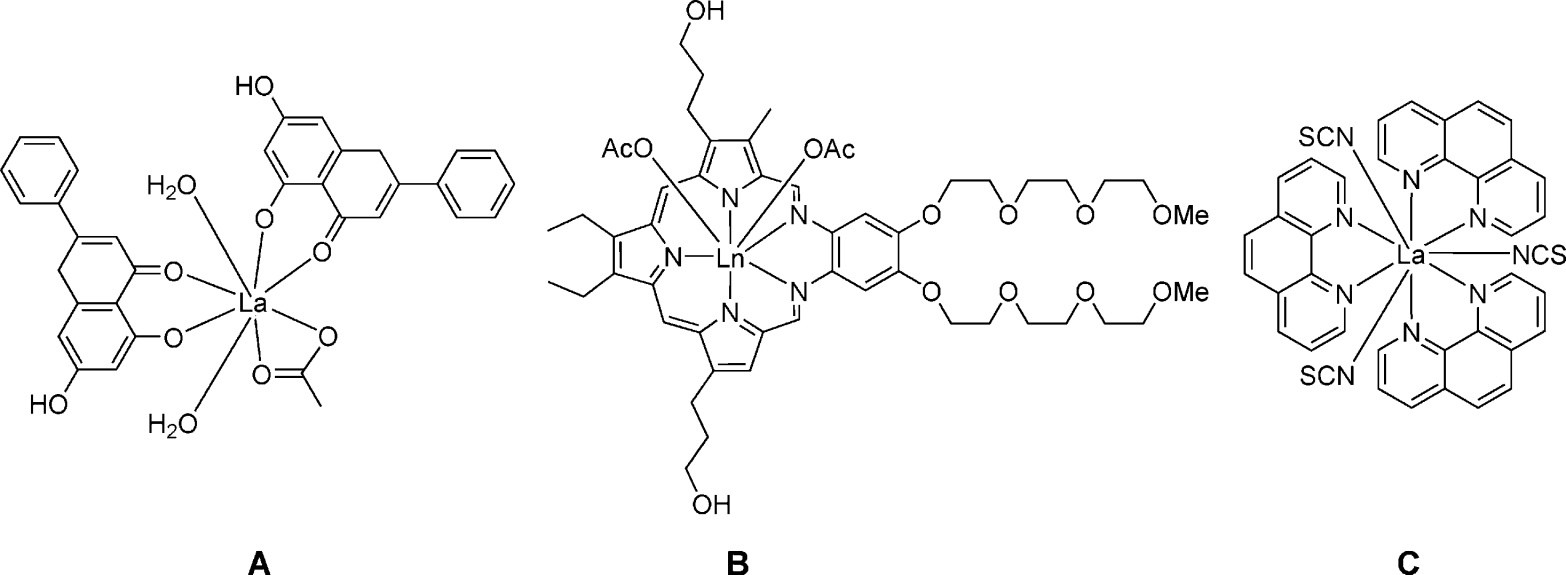


La(phen)_3_(NCS)_3_ (phen=1,10-phenanthroline) was found to overcome drug resistance and has proved to be highly effective against the DLD-1 colon cancer model in vivo,^26^–^28^ which is resistant to several chemotherapeutics amongst others due to oncogene mutations.^29^ Moreover, attempts to generate resistant to La(phen)_3_(NCS)_3_ cell models failed in contrast to many other investigated metallodrugs.

**Scheme 1:**
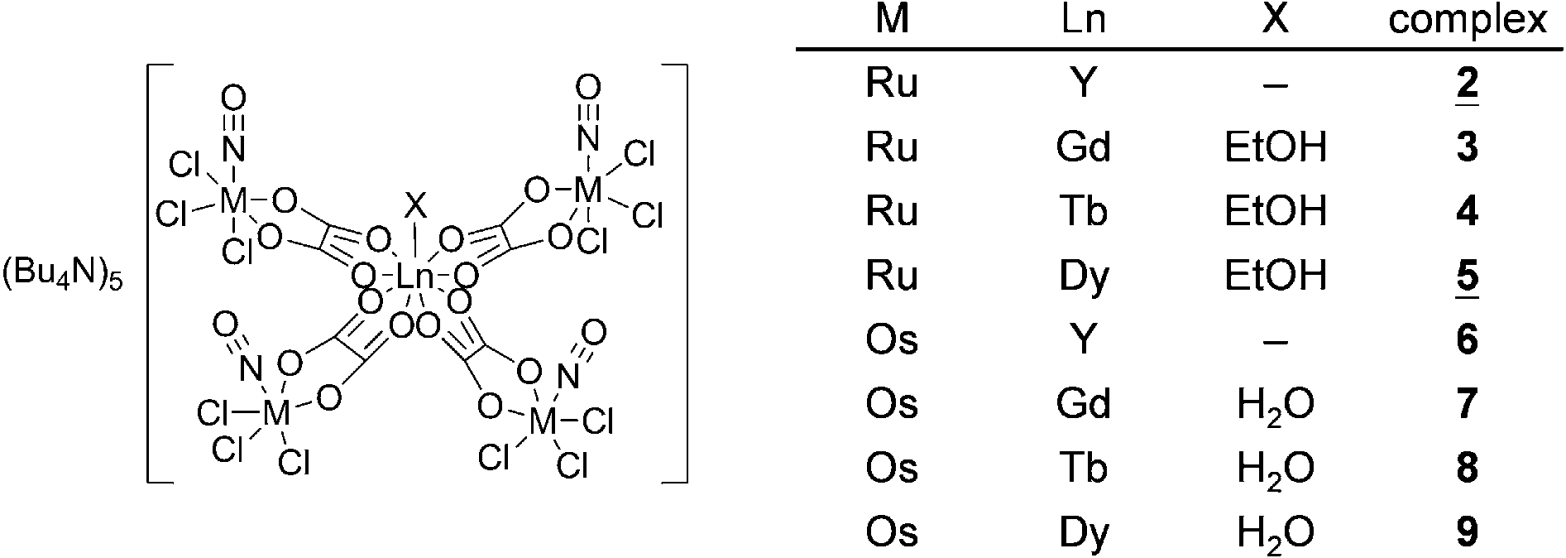
Compounds studied in this work. Underlined numbers indicate complexes investigated by X-ray diffraction. The synthesis of the osmium compounds 6–9 has been reported previously.^30^

Herein we report on the syntheses of lanthanide-labeled ruthenium–nitrosyl complexes as potential anticancer drugs. The lanthanide has been coupled to the ruthenium by a bridging oxalate, which is a well-known bioligand incorporated in the anticancer agent oxaliplatin.^31^ The complexes of the general formula (*n*Bu_4_N)_5_[Ln{RuCl_3_(ox)(NO)}_4_], where Ln=Y (**2**), Gd (**3**), Tb (**4**), Dy (**5**; Scheme 1), have been characterized by elemental analysis, ESI mass spectrometry and IR spectroscopy and, in case of **2** and **5** by X-ray diffraction. Their antiproliferative activity along with that of the precursor (*n*Bu_4_N)[RuCl_3_(μ-ox)(NO)] (**1**) have been investigated in the two human cancer cell lines HeLa (cervical cancer) and A549 (non-small cell lung cancer) and the noncancerous cell line MRC-5 (lung fibroblasts) and compared with that for the osmium analogues **6**–**9** (Scheme 1), which were reported recently.^30^

In addition, the effect of Ru versus Os as well as of the different lanthanide ions on the biological activity of the compounds is discussed and compared to that for the mononuclear ruthenium–nitrosyl complex **1**. The antiproliferative activity of Ru(NO)–Ln (4d–4f) and Os(NO)–Ln (5d–4f) complexes has been correlated with their accumulation in human A549 cancer cells.

## Experimental Section

### Materials

Solvents were obtained from commercial sources and were used as received. The starting compound Na_2_[RuCl_5_(NO)]⋅6 H_2_O was prepared as described in the literature.^32^ RuCl_3_⋅3 H_2_O was purchased from Johnson Matthey and the lanthanide salts were from Sigma–Aldrich. All chemicals were used as received. The respective osmium–lanthanide complexes **6**–**9** were synthesized as reported recently.^30^

### Synthesis of complexes

**(*n*Bu_4_N)_2_[RuCl_3_(ox)(NO)] (1)**: To a solution of Na_2_[RuCl_5_(NO)]⋅6 H_2_O (0.3 g, 0.6 mmol) in water (2.5 mL) a solution of oxalic acid (0.12 g, 1.3 mmol) in water (2.0 mL) was added. The pH value was adjusted to 3 using an aqueous solution of KOH. The reaction mixture was refluxed for 3 h and the pH of the solution was kept at 3. *n*Bu_4_NCl (0.36 g, 1.3 mmol) was added to the hot solution. The oil obtained was separated using a separation funnel and dissolved in water (20 mL). The solution generated purple crystals upon standing at room temperature. Yield: 0.20 g, 41 %; elemental analysis calcd (%) for C_34_H_72_Cl_3_N_3_O_5_Ru (*M*_r_=810.38 g mol^−1^): C 50.39, H 8.96, N 5.18; found: C 50.24, H 8.87, N 5.12; ESI-MS in MeOH (negative): *m*/*z* 568.9 [*M*−*n*Bu_4_N]^−^ (*m*_theor_=569.1); ESI-MS in MeOH (positive): *m*/*z* 241.8 [*n*Bu_4_N]^+^ (*m*_theor_=242.3); MIR (solid state ATR): 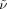
=2961, 2874, 1842 (NO), 1685, 1475, 1358, 1029, 887, 798, 745 cm^−1^; ^13^C NMR ([D_6_]DMSO, 125.82 MHz): *δ*=13.97, 19.58, 23.62, 58.06, 164.14, 166.64 ppm.

**(*n*Bu_4_N)_5_[Y{RuCl_3_(μ-ox)(NO)}_4_] (2)**: YCl_3_⋅3 H_2_O (13 mg, 0.043 mmol) was added to a solution of **1** (100 mg, 0.13 mmol) in acetonitrile (1.2 mL) and 2-propanol (0.65 mL) and the reaction mixture was refluxed for 1.5 h. The reaction mixture was cooled to room temperature and filtered. The solvent was removed under reduced pressure and the residue was dissolved in ethanol (2.0 mL). The product crystallized upon slow evaporation of the solvent at room temperature. Yield: 40 mg, 31 %; elemental analysis calcd (%) for C_88_H_180_Cl_12_N_9_O_20_Ru_4_Y (*M*_r_=2603.02 g mol^−1^): C 40.60, H 6.97, N 4.84; found: C 40.87, H 7.32, N 4.77; ESI-MS in MeOH (negative): *m*/*z* 568.4 (*n*Bu_4_N)[RuCl_3_(NO)(ox)]^−^ (*m*_theor_=569.1); ESI-MS in MeOH (positive): *m*/*z* 241.9 [*n*Bu_4_N]^+^ (*m*_theor_=242.3); MIR (solid state ATR): 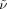
=2962, 2874, 1857 (NO), 1624, 1467, 1379, 1030, 883, 809, 739 cm^−1^; ^13^C NMR ([D_6_]DMSO, 125.82 MHz): *δ*=13.96, 19.69, 23.5, 58.04, 165.06, 167.56 ppm.

**(*n*Bu_4_N)_5_[Gd(EtOH){RuCl_3_(μ-ox)(NO)}_4_] (3)**: GdCl_3_⋅6 H_2_O (16 mg, 0.043 mmol) was added to a solution of **1** (100 mg, 0.13 mmol) in ethanol (2.0 mL) and the reaction mixture was refluxed for 1.5 h. The solution was cooled to room temperature and filtered. The solvent was removed under reduced pressure and the residue was dissolved in ethanol (2.0 mL). The product crystallized upon slow evaporation of the solvent at room temperature. Yield: 53 mg, 51 %; elemental analysis calcd (%) for C_90_H_186_Cl_12_GdN_9_O_21_Ru_4_⋅2 H_2_O for (*M*_r_=2753.48 g mol^−1^): C 39.18, H 6.94, N 4.57; found: C 38.95, H 7.07, N 4.58; ESI-MS in MeOH (negative): *m*/*z* 289.1 [RuCl_2_(NO)(ox)]^−^ (*m*_theor_=289.8), *m*/*z* 568.2 (*n*Bu_4_N)[RuCl_3_(NO)(ox)]^−^ (*m*_theor_=569.1), 1618.6 [*M*−3(*n*Bu_4_N)−[RuCl_3_(NO)(ox)]−EtOH]^−^ (*m*_*t*heor_=1618.9); ESI-MS in MeOH (positive): *m*/*z* 241.8 [*n*Bu_4_N]^+^ (*m*_theor_=242.3); MIR (solid state ATR): 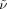
=2962, 2874, 1857 (NO), 1639, 1617, 1474, 1380, 1335, 884, 808, 738 cm^−1^.

**(*n*Bu_4_N)_5_[Tb(EtOH){RuCl_3_(μ-ox)(NO)_4_}] (4)**: TbCl_3_⋅6 H_2_O (16 mg, 0.043 mmol) was added to a solution of **1** (100 mg, 0.13 mmol) in ethanol (2.0 mL) and the reaction mixture was refluxed for 1.5 h. The solution was cooled to room temperature and filtered. The solvent was removed under vacuum and the residue was dissolved in ethanol (2.0 mL). The product crystallized upon slow evaporation of the solvent at room temperature. Yield: 41 mg, 44 %; elemental analysis calcd (%) for C_90_H_186_Cl_12_N_9_O_21_Ru_4_Tb (*M*_r_=2719.11 g mol^−1^): C 39.75, H 6.89, N 4.64; found: C 39.67, H 7.28, N 4.64; ESI-MS in MeOH (negative): *m*/*z* 569.0 (*n*Bu_4_N)[RuCl_3_(NO)(ox)]^−^ (*m*_theor_=569.1); ESI-MS in MeOH (positive): *m*/*z* 241.9 [*n*Bu_4_N]^+^ (*m*_theor_=242.3); MIR (solid state ATR): 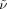
=2962, 2874, 1857 (NO), 1625, 1465, 1380, 882, 809, 739 cm^−1^.

**(*n*Bu_4_N)_5_[Dy(EtOH){RuCl_3_(μ-ox)(NO)}_4_] (5)**: DyCl_3_⋅6 H_2_O (16 mg, 0.043 mmol) was added to a solution of **1** (100 mg, 0.13 mmol) in acetonitrile (1.2 mL) and 2-propanol (0.65 mL) and the reaction mixture was refluxed for 1.5 h. The solution was cooled to room temperature and filtered. The solvent was removed under reduced pressure and the residue was dissolved in ethanol (2.0 mL). The product crystallized upon slow evaporation of the solvent at room temperature. Yield: 70 mg, 50 %; elemental analysis calcd (%) for C_90_H_186_Cl_12_DyN_9_O_21_Ru_4_ (*M*_r_=2722.67 g mol^−1^): C 39.70, H 6.89, N 4.63; found: C 39.71, H 7.21, N 4.64; ESI-MS in MeOH (negative): *m*/*z* 289.1 [RuCl_2_(NO)(ox)]^−^ (*m*_theor_=289.8), *m*/*z* 568.2 (*n*Bu_4_N)[RuCl_3_(NO)(ox)]^−^ (*m*_theor_=569.1), 1622.3 [*M*−RuCl_3_(ox)(NO)−3(*n*Bu_4_N)−EtOH]^−^ (*m*_theor_=1622.9); ESI-MS in MeOH (positive): *m*/*z* 241.7 [*n*Bu_4_N]^+^ (*m*_theor_=242.3); MIR (solid state ATR): 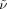
=2962, 1857 (NO), 1629, 1464, 1381, 1048, 881, 809, 739 cm^−1^.

### Physical measurements

Elemental analyses were performed by the microanalytical service of the Faculty of Chemistry of the University of Vienna on a Perkin–Elmer 2400 CHN Elemental Analyzer. UV/Vis spectra were recorded at 25 °C using a Perkin–Elmer Lambda 650 spectrometer equipped with an optical cell of 1 cm path-length in the wavelength range of 200 to 800 nm in combination with a Perkin–Elmer PTP-6 Peltier System. Electrospray ionization (ESI) mass spectrometry measurements were conducted on a Bruker HCT ion trap (Bruker Daltonics GmbH) by using methanol as a solvent. MIR spectra were recorded on a Perkin–Elmer 370 FTIR 2000 instrument using an ATR (attenuated total reflection) unit in the range of 4000–400 cm^−1^. Phosphorescence emission spectra were recorded with a Horiba FluoroMax-4 spectrofluorimeter and the data were processed using the FluorEssence v3.5 software package.

### X-ray crystallography

X-ray diffraction measurements were performed on a Bruker X8 APEXII CCD diffractometer. Single crystals were positioned at 35, 40 and 40 mm from the detector, and 950, 1964 and 4113 frames were measured, each for 10, 30 and 10 s over 1° scan width for **1**, **2** and **5**, respectively. The data were processed using SAINT software.^33^ Crystal data, data collection parameters, and structure refinement details are given in Table 1. The structures were solved by direct methods and refined by full-matrix least-squares techniques. Non-hydrogen atoms were refined with anisotropic displacement parameters. Hydrogen atoms were inserted in calculated positions and refined with a riding model. The following computer programs and hardware were used: structure solution, SHELXS-97 and refinement, SHELXL-97;^34^ molecular diagrams, ORTEP;^35^ computer, Intel CoreDuo. Disorder observed for tetrabutylammonium cation(s) in **2** and **5** was resolved by using SADI and EADP restraints and DFIX constraints implemented in SHELXL. CCDC 951636, 1402056 and 1402057 contain the supplementary crystallographic data for this paper. These data can be obtained free of charge from The Cambridge Crystallographic Data Centre.

**Table 1:** Crystal data and details of data collection for 1, 2 and 5.

Comp.	1	2	5
formula	C_34_H_72_Cl_3_N_3_O_5_Ru	C_88_H_180_Cl_12_N_9_O_20_Ru_4_Y	C_90_H_189_Cl_12_DyN_9_O_22.5_Ru_4_
*F*_w_	810.37	2603.00	2749.68
space group	*P* 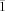	*P* 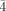 2_1_/*c*	*Cc*
*α* [Å]	11.8503(7)	18.4268(4)	17.9596(12)
*b* [Å]	18.0124(11)	18.4268(4)	27.2143(12)
*c* [Å]	21.2370(14)	18.4008(6)	27.0644(16)
*α* [°]	99.345(2)		
*β* [°]	104.935(3)		92.962(4)
*γ* [°]	98.681(2)		
*V* [Å^3^]	4232.5(5)	6247.9(3)	13 210.3(13)
*Z*	4	2	4
*λ* [Å]	0.71073	0.71073	0.71073
*ρ*_calcd_ [g cm^−3^]	1.272	1.384	1.383
size [mm^3^]	0.30×0.30×0.20	0.18×0.18×0.16	0.30×0.20×0.16
*T* [K]	150(2)	100(2)	100(2)
*μ* [mm^−1^]	0.598	1.247	1.307
*R*_1_^[a]^	0.0403	0.0278	0.0639
*wR*_2_^[b]^	0.1039	0.0746	0.1611
GOF^[c]^	1.033	1.052	1.129

[a] *R*_1_=Σ||*F*_o_|−|*F*_c_||/Σ|*F*_o_|. [b] *wR*_2_={Σ[*w*(*F*_o_^2^−*F*_c_^2^)^2^]/Σ[*w*(*F*_o_^2^)^2^]}^1/2^. [c] GOF={Σ[*w*(*F*_o_^2^−*F*_c_^2^)^2^]/(*n*−*p*)}^1/2^, where *n* is the number of reflections and *p* is the total number of parameters refined.

### XAS sample preparation

The Ru model compounds were diluted in BN (boron nitride, Sigma–Aldrich, CAS 10043-11-5, 99.5 %), filled into aluminum sample holders and sealed with Kapton foil. The BN samples were prepared for a calculated theoretical absorption of about 1 absorbance unit according to standards methods.^36^

### XAS data collection and analysis

The XAS experiment was carried out at beamline BM26A at the European Synchrotron Radiation Facility (ESRF) in Grenoble (France).^37^ At beamline BM26A (ESRF, Grenoble; France) three low noise ion chambers from Oxford Instruments were used for measurements in transmission mode. The absolute energy calibration was performed using a ruthenium powder (Sigma–Aldrich, CAS 7440-18-8, 99.9 %) BN preparation optimized for an absorption edge jump of 1 abs, measured at the same time between ionization chambers two and three. The model compound was measured in transmission mode. An Oxford CCC 1204 cryostat provided a sample environment of 20 K. The ESRF storage ring was operated at 6 GeV in the 7/8+1 filling mode. The beamline BM26A was equipped with a double crystal Si(111) monochromator and a bending magnet source giving an energy range of 5–30 keV (flux of 1×1011 ph s^−1^). Higher harmonics were rejected using two mirrors with Pt and Si coatings.

The XAS spectrum was measured at the Ru K-edge with a pre-edge region from 21 869 to 22 083 eV with a step size of 10 eV, an edge region from 22 099 to 22 161 eV with a step size of 1.3 eV. The k-space was measured from 3 to 14 A^−1^ with a step size of 0.05 A^−1^. The scanning times per measurement point were 1 s in the pre-edge, 5 s in the edge and 5–25 s (22 161–22 917 eV), increasing according to a predefined curve, in the post-edge region. The spectrum of the model compound is the average of 2 scans.

The program packages ATHENA,^38^ ARTEMIS,^38^ IFEFFIT,^39^ FEFF,^40^–^42^ PySpline,^43^ DL-EXCURV^44^–^47^ were applied for XAS data analysis.

XANES and EXAFS analysis and the interpretation applying the concept of the coordination charge have been performed as described recently.^48^

### Cell culture

Human cervical carcinoma (HeLa), human alveolar basal adenocarcinoma (A549), and normal human fetal lung fibroblast cell line (MRC-5) were maintained as monolayer culture in the Roswell Park Memorial Institute (RPMI) 1640 nutrient medium (Sigma Chemicals Co, USA). RPMI 1640 nutrient medium was prepared in sterile ionized water, supplemented with penicillin (192 IU mL^−1^), streptomycin (200 μg mL^−1^), 4-(2-hydroxyethyl)piperazine-1-ethanesulfonic acid (HEPES) (25 mM), L-glutamine (3 mM) and 10 % of heat-inactivated fetal calf serum (FCS; pH 7.2). The cells were grown at 37 °C in 5 % CO_2_ and humidified air atmosphere, by twice weekly subculture.

### MTT assay

Antiproliferative activity of the ruthenium and osmium complexes was determined using 3-(4,5-dimethylthiazol-yl)-2,5-diphenyltetrazolium bromide (MTT, Sigma–Aldrich) assay.^49^ Cells were seeded into 96-well cell culture plates (Thermo Scientific Nunc), at a cell density of 3000 cells per well (HeLa), 7000 cells per well (A549), and 5000 cells per well (MRC-5), in 100 μL of culture medium. After 24 h of growth, cells were exposed to the serial dilutions of the tested complexes. Complexes were dissolved in 1 % DMSO: complex **1** at a concentration of 4 mM, complexes **2**–**5** at a concentration of 1 mM, complexes **6**–**9** at a concentration of 0.6 mM, as stocks immediately prior use, and afterwards diluted with nutrient medium to desired final concentrations (in range up to 200 μM). Each concentration was tested in triplicate. After incubation periods of 48 h, 20 μL of MTT solutions (5 mg mL^−1^ in phosphate buffer solution, pH 7.2) were added to each well. Samples were incubated for 4 h at 37 °C, with 5 % CO_2_ in a humidified atmosphere. Formazan crystals were dissolved in 100 μL of 10 % sodium dodecyl sulfate (SDS). Absorbances were recorded after 24 h, on an ELISA reader (ThermoLabsystems Multiskan EX 200–240 V), at the wavelength of 570 nm. The IC_50_ values, defined as the concentrations of the compound causing 50 % cell growth inhibition, were estimated from the dose-response curves.

### Inductively coupled plasma mass spectrometry (ICP-MS)

**Sample preparation for the measurement of intracellular Ru/Os accumulation using ICP-MS**: Ru/Os accumulation was analyzed in A549 cells with ICP-MS using Thermo Scientific iCAP Qc ICP-MS (Thermo Scientific, Bremen, Germany).^50^ A549 cells were seeded into a 25 cm^2^ dish (Thermo Scientific Nunc™) and treated with the complexes **4** and **8** at concentrations equal to 0.5×IC_50_. After 6 and 24 h, cells were harvested by scraping, washed with ice-cold PBS and collected by centrifugation at 778 g for 10 min.

**Sample preparation for the measurement of Ru/Os binding to DNA and proteins using ICP-MS**: Binding of Ru/Os to cellular DNA and proteins was analyzed in A549 cells, using ICP-MS. A549 cells were prepared and collected using the same procedure as described above. Total DNA and protein were isolated using TRI Reagent® (Sigma–Aldrich) according to the manufacturer’s procedure and concentrations were determined spectrophotometrically by measuring absorbances (Eppendorf BioPhotometer 6131).

### Microwave digestion

The digestion of the samples for ICP-MS studies was performed on an advanced microwave digestion system (ETHOS 1, Milestone, Italy) using HPR-1000/10S high pressure segmented rotor. The pressure-resistant PTFE vessels (volume 100 mL) used in this study consisted of fluoropolymer liner. Before use, the PTFE vessels were acid cleaned and rinsed with deionized water. This type of vessel permitted a maximum temperature of 240 °C and a maximum pressure of 100 bar to be applied. Maximally ten PTFE vessels could simultaneously be mounted on the rotor. The internal temperature was monitored only with one vessel equipped with a sensor unit, and this vessel had a sensor-protecting tube that directly contacted the digested solution, differing from the other common PTFE vessels. In the digestion, samples were mixed in each clean vessel with 4 mL HNO_3_ (65 %, Suprapure, Merck, Germany) and 4 mL ultrapure water and then heated with microwave energy for 10 min. The temperature was controlled with a predetermined power program. Digestion of the samples was carried out for 10 min at a constant temperature of 180 °C, with a prior warm-up linearly over 10 min to 180 °C. After cooling and without filtration, the solution was diluted to a fixed volume into a 10 mL volumetric flask and made up to volume with ultrapure water. Ultrapure water was prepared by passing doubly deionized water from Milli-Q system (Millipore, Bedford) to a resistivity of 18.2 MΩ cm.

### Instrumental analysis

ICP-MS measurements were performed using Thermo Scientific iCAP Qc ICP-MS (Thermo Scientific, Bremen, Germany) spectrometer with operational software Qtegra. For Ru determination the instrument was adjusted for optimum performance in He KED (kinetic energy discrimination) mode using the supplied autotune protocols. For Os determination the instrument was adjusted for optimum performance standard no gas mode using the supplied autotune protocols. The instrumental operating conditions for ICP-MS are shown in Table 2.

**Table 2:** Experimental conditions used on ICP-MS equipment to determine Ru and Os in samples.

Parameter	Experimental conditions
radio frequency power (RF) nebulizer argon flow rate auxiliary argon flow rate Coolant argon flow rate CCT1-Helium dwell time extraction Sample uptake rate spray chamber nebulizer number of readings per replicate software Isotopes	1550 W 0.95 L min^−1^ 0.80 L min^−1^ 14.0 L min^−1^ 6.0 mL min^−1^ 10 ms −5000 V 0.40 mL min^−1^ cyclonic Meinhard ESI MicroFlow PFA-ST 3 Qtegra ^101^Ru, ^189^Os

Analytical blanks were run in the same way as the samples, and concentrations were determined using standard solutions prepared in the same acid matrix. The standard for the instrument calibration was prepared on the basis of ruthenium, plasma standard solution, Specpure®, Ru 1000 μg mL^−1^ and osmium, plasma standard solution, Specpure, Os 1000 μg mL^−1^ certified reference solutions ICP standard purchased from Alfa Aesar GmbH & Co KG (Germany).

### Abbreviations

XAS, X-ray absorption spectroscopy; XANES, X-ray absorption near edge structure; EXAFS, extended X-ray absorption fine structure; ESRF, European Synchrotron Radiation Facility; FT, Fourier transform.

## Results and Discussion

### Synthesis and characterization

As mentioned in the Introduction we were interested in labeling our ruthenium and osmium–nitrosyl complexes with lanthanide ions. A suitable strategy to accomplish such a combination was inspired by a previous report on a heteropentanuclear oxalate-bridged [Re^IV^_4_Gd^III^] complex.^51^ Quite recently we reported the synthesis of the osmium–nitrosyl analogues **6**–**9** (Scheme 1).^30^ The ruthenium–nitrosyl analogues **2**–**5** (Scheme 1) were synthesized following a similar procedure. An aqueous solution of Na_2_[RuCl_5_(NO)] was treated with 2 equiv oxalic acid to give rise to [RuCl_3_(ox)(NO)]^2−^, which was isolated as a tetrabutylammonium salt **1** in 47 % yield by addition of *n*Bu_4_NCl to the reaction mixture. This complex proved to be suitable for the synthesis of pentanuclear heterometallic complexes **2**–**5** by treatment with 0.3 equiv of the respective lanthanide(III) or yttrium(III) salt in 4:1 molar ratio either in ethanol or in a mixture of acetonitrile and 2-propanol. All these 4d–4f metal complexes were obtained as crystalline solids in 31–51 % yield. The formation of pentanuclear assemblies was confirmed by elemental analysis, ESI mass spectrometry and X-ray diffraction of complexes **2** and **5**. ESI mass spectra of complexes **1**, **2**, and **5** showed only a signal for the ruthenium fragment [RuCl_3_(NO)(ox)]^−^ (*m*/*z* 569) in the negative ion mode, while for **3** and **4** signals with a higher *m*/*z* ratio were observed, which could be attributed to tetranuclear species containing the respective lanthanide ion. ^13^C NMR spectroscopic measurements of the diamagnetic compound **2** revealed no significant influence of the Y^III^ coordination on the chemical shifts of the oxalato carbon atoms.

### Photophysical properties

The luminescence of the reported compounds was investigated using complex **4** as an example, since this property is most evident and often studied for europium or terbium ions. Emission spectra (*λ*_ex_=365 nm) of aqueous solutions of **4** at different concentrations ranging from 0.1 to 400 mM were measured between 450 and 700 nm using a slit width of 10 nm. Even at the highest concentration level, only a very weak phosphorescence signal was found after ten flash counts (data not shown). To be able to observe the complete emission pattern typical for a terbium ion consisting of four peaks, up to 200 flash counts had to be applied (Figure S1 in the Supporting Information). These attempts indicate that complexes of the type **2**–**5** are not suitable for further development as potential diagnostic agents.

### X-ray diffraction analysis

**Figure 1:**
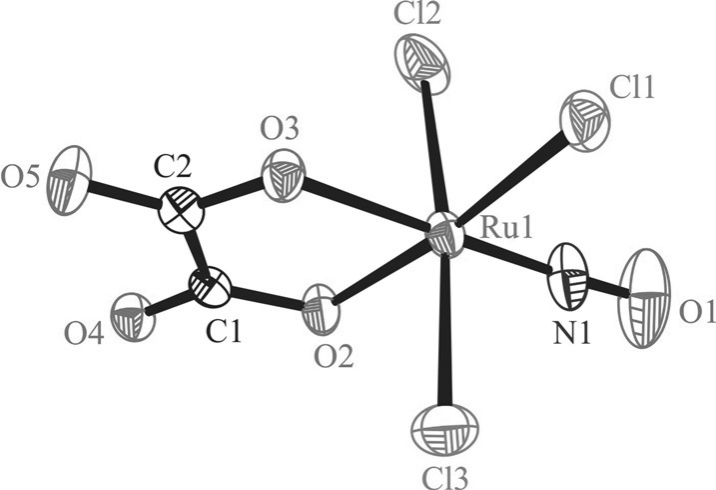
ORTEP view of the complex anion [RuCl_3_(ox)(NO)]^2−^. Thermal ellipsoids are drawn at the 50 % probability level. Selected bond lengths [Å] and bond angles [°]: Ru1−Cl1 2.3482(7), Ru1−Cl2 2.3599(7), Ru1−Cl3 2.3674(7), Ru1−N1 1.714(2), Ru1−O2 2.0440(18), Ru1−O3 2.0037(16), N1−O1 1.150(3); Ru1-N1-O1 177.0(3), O2-Ru1-O3 80.73(7).

**Figure 2:**
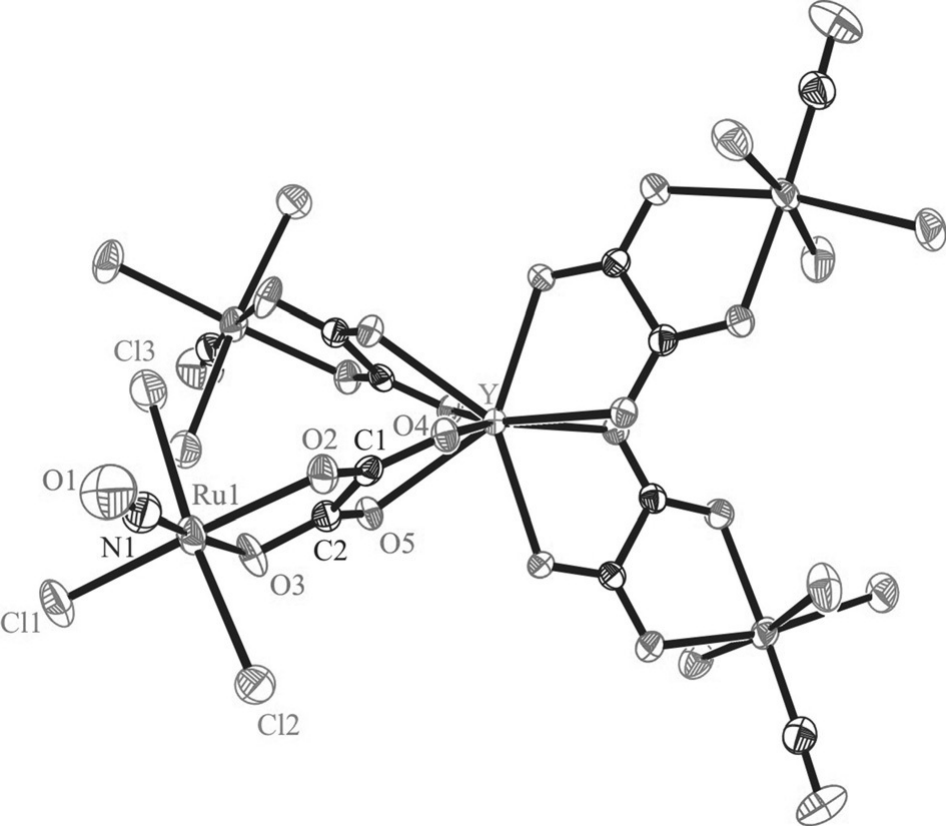
ORTEP view of the complex anion [Y{RuCl_3_(μ-ox)(NO)}_4_]^5−^. Thermal ellipsoids are drawn at the 50 % probability level. Selected bond lengths [Å] and bond angles [°]: Ru1−Cl1 2.3202(9), Ru1−Cl2 2.3642(10), Ru1−Cl3 2.3412(10), Ru1−N1 1.716(3), Ru1−O2 2.075(2), Ru1−O3 2.046(2), Y−O4 2.329(2), Y−O5 2.387(2), N1−O1 1.151(4); Ru1-N1-O1 176.8(3), O2-Ru1-O3 80.24(9), O4-Y-O5 69.41.

**Figure 3:**
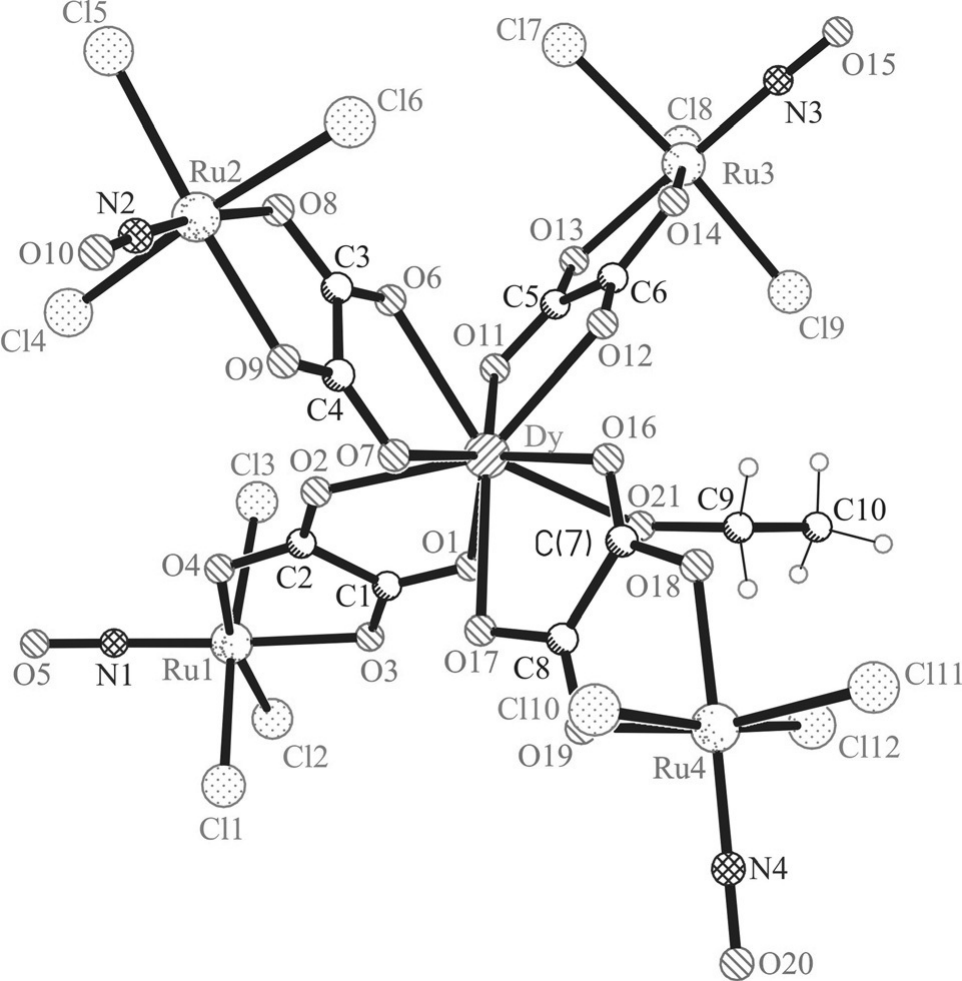
Ball and stick view of the complex anion [Dy(EtOH){RuCl_3_(μ-ox)(NO)}_4_]^5−^.

The results of X-ray diffraction studies of complexes **1**, **2** and **5** are shown in Figure 1, Figure 2 and Figure 3. Note that the X-ray diffraction structure of [RuCl_3_(ox)(NO)]^2−^ was reported previously as a cesium salt and [NiL]^2+^ complex, where L=1,4,8,11-tetraazacyclotetradecane.^52^ In the present work the complex (*n*Bu_4_N)_2_[RuCl_3_(ox)(NO)] (**1**) was studied since it served as a starting material for further complexation reactions with lanthanide salts. In addition, we used this compound for determination of the oxidation state of ruthenium coordinated to a non-innocent NO ligand (vide infra). In **2** four complex anions [RuCl_3_(ox)(NO)]^2−^ are coordinated to yttrium(III) via unbound oxygen atoms of the oxalates which act as bridging ligands with formation of the complex [Y{RuCl_3_(μ-ox)(NO)}_4_]^5−^. The negative charge is counterbalanced by five *n*Bu_4_N^+^ ions present in the crystal structure. Like (*n*Bu_4_N)_5_[Y{OsCl_3_(μ-ox)(NO)}_4_]^30^ the complex crystallizes in the tetragonal space group *P*
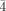
2_1_*c*. The yttrium atom and the nitrogen atom of one of the tetrabutylammonium cations lie on the fourfold rotation axis running along the *c* axis. So the asymmetric unit consists of one [RuCl_3_(ox)(NO)]^2−^ unit bound to Y^III^ in a special position, one *n*Bu_4_N^+^ cation in a general position and a quarter of *n*Bu_4_N^+^ in a special position. The whole complex forms a sphere the radius of which is of approximately 8.5 Å. The shortest Ru⋅⋅⋅Ru separation is of 7.469 Å, while Y⋅⋅⋅Y distance is of 15.951 Å.1

Selected bond lengths and bond angles in the coordination spheres of Ru and Y are quoted in the legend to Figure 2. The Ru−Cl bonds in **2** are well-comparable to those in the precursor **1**, while the Ru−O bonds are by approximately 0.03–0.04 Å longer in **2** compared to those in **1**. The Ru−N1 bond in **2** is very similar to that in **1**, as also are the N1−O1 bond and the Ru1-N1-O, 1 angle in both complexes.

The complex (*n*Bu_4_N)_5_[Dy(EtOH){RuCl_3_(μ-ox)(NO)}_4_]⋅1.5 H_2_O crystallizes in the monoclinic non-centrosymmetric space group *Cc*. The whole structure is severely affected by the disorder, which was mainly resolved in an isotropic model. Therefore, a comparison of the metric parameters in **1** with those in **5** has not much sense. The asymmetric unit consists of one complex anion [Dy(EtOH){RuCl_3_(μ-ox)(NO)}_4_]^5−^ and five *n*Bu_4_N^+^ cations. While Y^III^ is eight-coordinate in **2**, the Dy^III^ ion is nine-coordinate in **5** with an additional coordination of an EtOH molecule, as was also found for the corresponding Os–Dy counterpart.^30^ Complexes **3** and **4** were found to crystallize in the monoclinic non-centrosymmetric space group *Cc*, and are affected by severe disorder. Therefore, only the parameters of the unit cells are given in Table S1 in the Supporting Information for the two compounds. In addition, crystallization of **4** from a CHCl_3_ solution yielded crystals isostructural to that of **2** (see Table S1, entry 4’ for the respective unit-cell parameters).

NO in complexes **1**–**5** acts as a non-innocent ligand^53^ rendering the description of the exact electronic structure of the Ru(NO) entity difficult. According to Enemark and Feltham notation^54^ and taking into account the diamagnetism of **1**, the close to linearity Ru-N-O angle and the IR *ν*(NO) vibration (1842 cm^−1^) it can be described in our case as {Ru(NO)}^6^. However, it does not reveal the actual physical and formal oxidation state^55^ of the ruthenium and NO ligand. Therefore, XANES experiments were performed to determine the physical oxidation state of ruthenium in **1**.

### XAS analysis

The XAS spectra of the Ru reference compounds have been published recently.^48^ The method has been proven highly valuable for determining the oxidation state and coordination charge for metal complexes in vivo and in vitro.^56^, ^57^

In this study they formed the basis for the oxidation state assignment of ruthenium in **1**. The structural formulas are shown in Figure S2 in the Supporting Information. The prefix “R” has been added to the labeling and numbering of the reference compounds is preserved to avoid overlaps. The figure includes the following model compounds: indazolium *trans*-[tetrachloridobis(1*H*-indazole)ruthenate(III)] **R1** (with first coordination shell Ru^III^Cl_4_N_2_),^58^ tris(pentan-2,4-dionato)ruthenium(III) (**R3**, Ru^III^O_6_, Sigma–Aldrich, CAS 14284-93-6, 97 %),^59^ hexammineruthenium(III) trichloride (**R4**, Ru^III^N_6_, Sigma–Aldrich, CAS 14282-91-8, 99 %),^60^ *mer*,*trans*-aquatrichloridobis(indazole)ruthenium(III) (**R5**, Ru^III^Cl_3_N_2_O),^61^ *trans*,*trans*-dichloridotetrakis-(indazole)ruthenium(III) chloride (**R6**, Ru^III^Cl_2_N_4_),^62^ *mer*-trichloridotris(indazole)-ruthenium(III) (**R7**, Ru^III^Cl_3_N_3_),^63^ hexammineruthenium(II) dichloride (**R8**, Ru^II^N_6_, Sigma–Aldrich, CAS 15305-72-3, 99.9 %),^64^ *mer*,*trans*-trichlorido(dimethylsulfide)bis(indazole)-ruthenium(III) (**R9**, Ru^III^Cl_3_N_2_S),^65^ *trans*,*trans*- dichloridotetrakis(indazole)ruthenium(II) (**R10**, Ru^II^Cl_2_N_4_),^62^ *mer*-trichloridotris(ethylphenylsulfide)ruthenium(III) (**R11**, Ru^III^Cl_3_S_3_),^66^ *trans*,*trans*,*trans*-dichloridobis(dimethylsulfide)bis(indazole)ruthenium(II) (**R12**, Ru^II^Cl_2_N_2_S_2_).^62^

**Figure 4:**
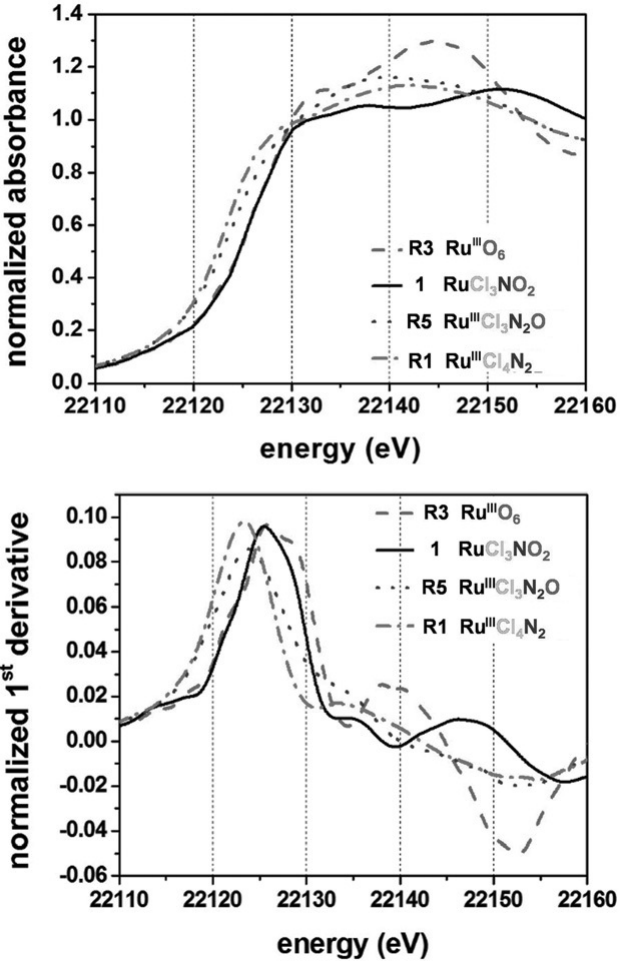
Normalized XANES spectra of 1 and the model compounds R1, R3, and R5 (top), and their corresponding first derivatives (bottom).

The XANES spectra and their corresponding first derivatives for **1**, **R1** (first shell coordination: Ru^III^Cl_4_N_2_), **R5** (Ru^III^Cl_3_N_2_O) and **R3** (Ru^III^O_6_) exhibiting a mixed chloride, nitrogen/oxygen first coordination sphere are shown in Figure 4. The edge position for **1** (RuCl_3_NO_2_) was determined as 22 125.9 eV. In comparison to **R7** (Ru^3+^Cl_3_N_3_) and **R5** (Ru^3+^Cl_3_N_2_O), **1** displays an edge shift of +2.2 and +1.9 eV, respectively. The edge position for **R3** (Ru^III^O_6_) has been determined to be 22 126.6 eV and appears 0.7 eV above that for **1**. In the previous study on ruthenium complexes it was shown that model compounds with the same first shells show an edge shift of about +2 eV on going from Ru^II^ to Ru^III^.^48^ Like models **R1**, Ru^III^Cl_4_N_2_, **R3**, Ru^III^O_6_, and hexammine compounds **R8**, Ru^II^N_6_ and **R4**, Ru^III^N_6_ complex **1** (RuCl_3_NO_2_) exhibits a characteristic edge shoulder.^48^,4

**Figure 5:**
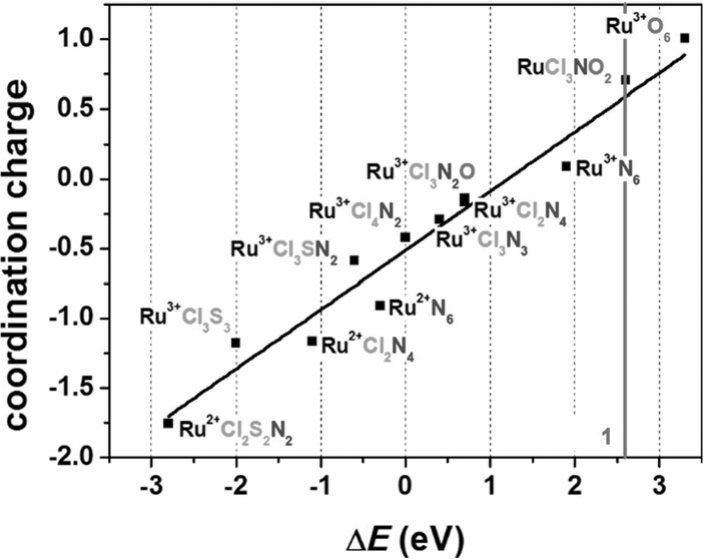
The calculated coordination charge *η*_AR_ according to the Allred–Rochow scale in comparison to the observed edge energies of the XANES spectra. The edge energy of R1 Ru^III^Cl_4_N_2_ in boron nitride (BN) was set as an arbitrary origin.

In Figure 5 the calculated coordination charges versus the experimental determined Ru K-edge positions are shown. The edge energy of **R1** Ru^III^Cl_4_N_2_ in boron nitride (BN) was set as an arbitrary origin. A regression line with a coefficient of determination *R*^2^=0.95 could be aligned to the calculated coordination charges and the observed edge positions of Ru^II^ and Ru^III^ model compounds with varying absorber–ligand environments, thereby proving the linear correlation between the coordination charge and the edge positions.^48^ The compounds containing Ru^II^ and/or S are on the left (lower energy) side, and the compounds containing Ru^III^, N, and O are on the right (higher energy) side. The edge position of 22 125.9 eV for (*n*Bu_4_N)_2_[RuCl_3_(ox)(NO)] clearly falls in the range of Ru^3+^ compounds with a mixed nitrogen/oxygen/chloride coordination sphere.5

**Figure 6:**
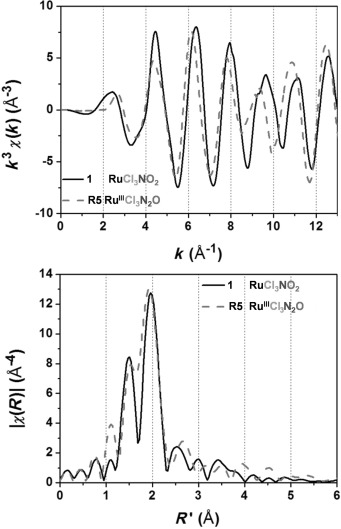
The *k*^3^-weighted extracted fine structures (top) and Fourier transforms (bottom) of 1 and the model compound R5.

The *k*^3^-weighted EXAFS spectra and the Fourier transforms (FT) of **1** are shown in Figure 6. For compounds with mixed N/O/Cl first shells and increasing number of N and/or O ligands a splitting of the first peak in the FT is observed. The backscattering amplitudes of the heavy scatterers like S and Cl and the light scatterers, like N and O are out of phase.^67^ For **1** this cancellation leads to a node between 8 and 11 Å^−1^ shown in Figure 6 (top; black curve). The fitting analysis using FEFF^41^, ^42^ was restricted to the first coordination shell extracted from the first peak in the FT. The identity and numbers of back-scatterers were fixed not to exceed the number of fitting parameters and the known crystallographic distances were taken as a starting point for the fitting analysis.6

The results of the first shell fit of **1** using theoretical amplitudes and phases provided by the FEFF code are presented in Table 3, as well as the results for the DL-EXCURV fit (Table 4).^47^ The distances are given as the average fitted distances for each atom type/shell (Cl, O/N and N/C). The curve fitting results obtained by FEFF and DL EXCURVE are both in good agreement with the crystallographic values.

**Table 3:** First-shell fit of the model compounds using theoretical amplitudes and phases provided by the FEFF code.^[a]^

Comp.	Path	*N*_fix_	*R* [Å]	*R*_cryst_ [Å]	Δ*R* [Å]	*σ*^2^ [Å^2^×10^−3^]	*E*_0_ [eV]	Fit index [%]
**1**	Ru-N	1	1.74(2)	1.714	0.026	1.73±0.36	6.1±1.0	0.7
	Ru-O	2	2.07(3)	2.024	0.045			
	Ru-Cl	3	2.39(3)	2.358	0.032			

[a] *N*_fix_ is the fixed coordination number, *R* is the average distance, *R*_cryst_ is the crystallographic value, Δ*R* is the difference between *R* and *R*_cryst_, *σ*^2^ is the Debye–Waller factor, *E*_0_ is the residual shift of the edge energy.

**Table 4:** DL-EXCURV EXAFS fit of the model compounds.^[a]^

Comp.	Path	*N*_fix_	*R* [Å]	*R*_cryst_ [Å]	Δ*R* [Å]	*σ*^2^ [Å^2^×10^−3^]	*E*_0_ [eV]	*R*_fit_ [%]	Fit index [%]
**1**	Ru−N	1	1.753(7)	1.714	0.039	1.44±0.80	−4.7±0.6	18.66	0.24
	Ru−O	2	2.032(6)	2.024	0.008	2.25±0.24			
	Ru−Cl	3	2.374(3)	2.358	0.016				
	Ru−C	2	2.73(2)	2.826	−0.096	2.56±1.76			

[a] *N*_fix_ is the fixed coordination number, *R* is the average distance, *R*_cryst_ is the crystallographic value, Δ*R* is the difference between *R* and *R*_cryst_, *σ*^2^ is the Debye–Waller factor, *E*_0_ is the residual shift of the edge energy, *R*_fit_ is the quality of the fit, fit index is the sum of the square of the residuals.

The only other Ru–NO compound, the oxidation state of which has been investigated by XANES spectroscopy, is *mer*,*trans*-[RuCl_3_(1*H-*indazole)_2_(NO)].^32^ The authors compared the edge position of *mer*,*trans*-[RuCl_3_(1*H-*indazole)_2_(NO)] with the one of **R5** (Ru^3+^Cl_3_N_2_O), and **R10** (Ru^2+^Cl_2_N_4_) and concluded the oxidation state of 3.4(3)+ for Ru in *mer*,*trans*-[RuCl_3_(1*H-*indazole)_2_(NO)]. The oxidation state of 3.4+ might be slightly overestimated owing to the lack of data for Ru^IV^ reference compounds indicating that the physical oxidation number,^53^, ^55^ which is a measurable quantity derived from a known d^*n*^ configuration of a metal ion, in that case is 3+ (d^5^ electron configuration). It differs from the formal oxidation state 4+ or 2+ of ruthenium in this mononuclear complex, which is a non-measurable integer defined as the charge left on the metal after all ligands in [RuCl_3_(1*H-*indazole)_2_(NO)] have been removed in their normal, closed-shell configuration.^55^ In the case of non-innocent ligand NO its closed shell configurations can be represented as NO^−^ or NO^+^.

### Stability in aqueous media

The stability of the heteropentanuclear complexes **5** and **9** in aqueous solution has been investigated by UV/Vis spectroscopy over 96 h. There was no change in the optical spectra observed (Figures S3 and S4 in the Supporting Information), which indicates a high stability of the complexes under applied conditions.

To exclude immediate dissociation of the pentanuclear assembly with release of a metal–oxalate fragment an aqueous solution of **5** was evaporated to dryness after standing for 24 h in air and the IR spectra of the residue and freshly prepared compound **5** were compared. There were no differences in the spectra observed.

### Cytotoxic activity

The antiproliferative activity of the ruthenium and osmium lanthanide complexes **1**–**9** was evaluated for 48 h of continuous drug action, using colorimetric MTT assay. The study was performed in two human neoplastic cell lines (HeLa, A549), and human fetal lung fibroblast cell line (MRC-5), which was used as noncancerous model for in vitro toxicity evaluation. The results are shown in Table 5 in terms of IC_50_ values for 48 h incubation period. IC_50_ values are calculated as mean values obtained from two to three independent experiments and are presented with their standard deviations.

**Table 5:** Results of MTT assay presented as IC_50_ [μM] values obtained after 48 h treatment.^[a]^

IC_50_ [μM]				
	HeLa (3000 c per w)	A549 (7000 c per w)	MRC-5 (5000 c per w)
1	55.9±5.6	55.0±1.6	18.1±6.5
2	17.9±0.4	56.3±1.1	12.0±0.3
3	29.1±0.8	26.5±4.0	11.1±0.1
4	20.0±2.4	22.4±3.1	11.9±0.4
5	20.0±2.1	47.0±6.7	13.2±0.4
6	152.1±24.0	>200	114.9±10.7
7	152.7±19.9	>200	151.1±0.7
8	118.1±2.9	>200	133.3±3.5
9	147.2±3.5	>200	139.9±6.8

[a] IC_50_ values are calculated as mean values obtained from three independent experiments and quoted with their standard deviations; c per w=cells per well.

The results showed that all tested ruthenium compounds exhibited dose-dependent cytotoxicity in the range of concentrations up to 200 μM, being up to 10-times more active than their osmium analogues, especially in A549 cells, where osmium compounds did not reach their IC_50_ values in the examined range of concentrations. Concentration-effect curves for each cell line are depicted in Figure S5 in the Supporting Information, illustrating the pronounced differences in activity between complexes containing ruthenium and osmium. These differences are of special note, since osmium compounds were reported to be either as active as or even more potent than their ruthenium analogues.^10^, ^12^, ^68^–^70^ Exceptions have only been reported for a pair of Ru^III^/Os^III^ tetrazole complexes^13^ and a series of ruthenium and osmium complexes containing azole and NO ligands,^1^ where the ruthenium complexes were found to be significantly more active.

Higher IC_50_ values obtained after treatment of A549 compared to HeLa cells were expected, because of decreased sensitivity and slower response to treatment of A549 cells. Complex **4** exhibited the highest antiproliferative activity in general, with that against A549 cells in the range of activity obtained in HeLa cells (20.0(±2.4) vs. 22.4(±3.1) μM, respectively), which is a promising result.

Cell-type selectivity is also noted in the analysis of the effect of tested compounds (ruthenium and osmium analogues) in MRC-5 normal cell line. While osmium compounds exhibited cytotoxic activity in MRC-5 comparable to cytotoxicity in HeLa cells, ruthenium complexes showed high cytotoxic potential in vitro in MRC-5 cell line, which may be considered as the major drawback in the preliminary studies of these complexes.

Comparison of antitumor activity of **2**–**5** with that of **1** indicates slightly higher activity of the former species. Dissociation of **2**–**5** with release of Ln^III^ would generate 4 equiv of **1** and approximately a fourfold increase of cytotoxicity would be expected. Indirectly, these data provide further evidence about the stability of the complexes under the conditions used for MTT assays. Enhancement of antiproliferative activity by coordination of organic biologically active species to the Ln^III^ ion is well-documented in the literature.^71^

### Intracellular distribution/accumulation of investigated complexes

Discovery and development of new metal-based anticancer agents is largely based on cell viability assays (IC_50_ values), intracellular accumulation and distribution studies. Considering the obtained IC_50_ values, complexes **4** and **8** were chosen for the ICP-MS analysis in order to investigate intracellular distribution and accumulation of Ru/Os in A549 cells.

**Figure 7:**
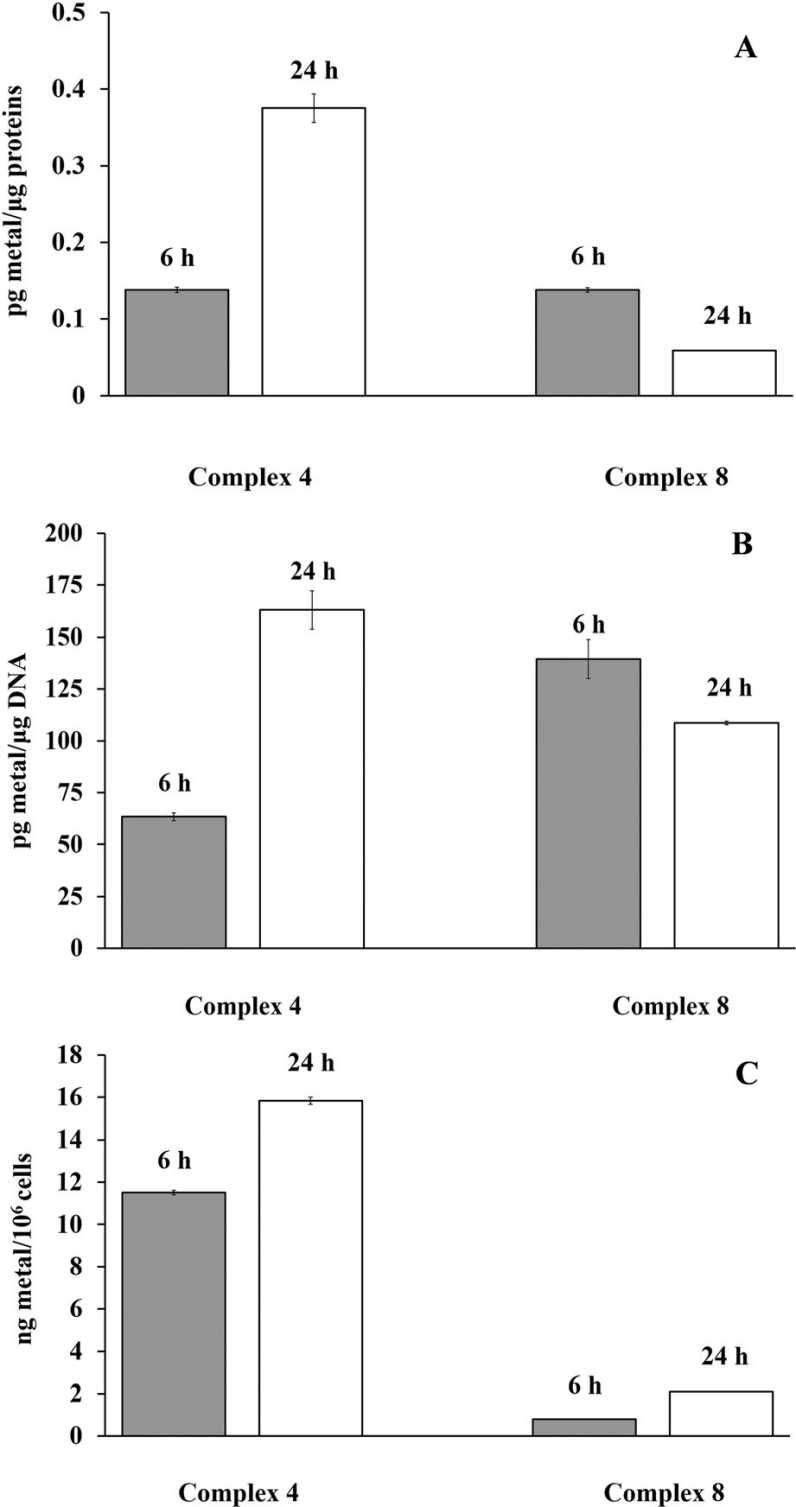
Ru/Os content in A549 cells after 6 and 24 h, measured by ICP-MS. A) Total intracellular accumulation of investigated metals. B) Metal content in DNA fraction of cells. C) Metal content in protein fraction of cells. All experiments were performed in triplicate and presented with corresponding standard deviations.

In particular, we separately analyzed metal (Ru/Os) distribution among the DNA and protein fractions, as well as total intracellular accumulation, using ICP-MS analysis, after 6 and 24 h treatment with 0.5×IC_50_ of the investigated complexes. Each analyzed metal compound was found in the cells, although exhibiting different levels of accumulation and various affinities for protein and DNA binding. Ruthenium exhibited greater (five- to eightfold) total intracellular accumulation than osmium, with time-dependent increase of accumulation characteristic for both metals (Figure 7 A). The results also show that osmium complex **8** was bound to cellular DNA more efficiently than Ru complex following 6 h treatment (139.5(±9.4) vs. 63.4(±1.8) pg metal μg^−1^ DNA, respectively). With prolonged incubation time, complex **4** induced more DNA binding, while complex **8** exhibited the opposite behavior, although at the lesser extent (Figure 7 B). Analyses of metal content in protein cell fractions indicated that after 6 h treatment both complexes induce a similar level of metal binding to cellular proteins, but with the treatment prolongation the level of Os–protein binding decreased twofold, while Ru–protein binding increased to a great extent (Figure 7 C).7

Low Os intracellular accumulation compared to Ru, as well as time-dependent decrease of protein/DNA binding, indicate a reversible nature of interactions of complex **8**, which all may be a reason for its low cytotoxic action. Higher cytotoxicity of **4** compared to **8** may be attributed to its ability to bind DNA and proteins more efficiently, to increase the number of interactions with these biomolecules over time, and possibly to form different DNA and protein conformational distortions and lesions. As a result of these differences in cellular accumulation and DNA/protein binding, potential differences in cellular response to Ru/Os treatment arise.

## Conclusions

Following our interest in ruthenium and osmium nitrosyl compounds with promising biological properties, we have successfully synthesized a series of pentanuclear ruthenium lanthanide complexes of the general formula (*n*Bu_4_N)_5_{Ln[RuCl_3_(NO)(μ-ox)]_4_(EtOH)_*n*_} (*n*=0 for Ln=Y (**2**), *n*=1 for Ln=Gd (**3**), Tb (**4**), Dy (**5**)) from the treatment of (*n*Bu_4_N)_2_[RuCl_3_(ox)(NO)] (**1**) with the respective lanthanide salt in ethanol or in acetonitrile/2-propanol mixture.

Investigations on the luminescence properties of the reported compounds revealed only a very weak phosphorescence signal for the terbium complex **4**, rendering this compound class unsuitable as theranostic agents. Nevertheless, the idea in our opinion deserves further attention and can be verified when using other types of *n*d–4f metal complexes with more appropriate organic ligands.

Detailed analysis of the in vitro antitumor activity concerning complexes **2**–**5** showed a cytotoxicity enhancement of the synthesized compounds compared to the starting compound (**1**). Lower activity of the previously reported osmium analogues **6**–**9** in all tested cell lines leads to the conclusion that the presence of the ruthenium center in heteronuclear *n*d–4f metal complexes enhances their cytotoxicity. Moreover, the cytotoxic potential of investigated complexes **2**–**5** is in accord with a five- to eightfold greater cellular accumulation of ruthenium compared to osmium obtained from ICP-MS investigations of complexes **4** and **8**. Further studies on elucidating mechanisms underlying different anticancer activity and cellular accumulation of these compounds are required in order to ascertain their potential for the development as anticancer agents.

## Supporting Information

As a service to our authors and readers, this journal provides supporting information supplied by the authors. Such materials are peer reviewed and may be re-organized for online delivery, but are not copy-edited or typeset. Technical support issues arising from supporting information (other than missing files) should be addressed to the authors.

miscellaneous_information
