# Supplementary material for: Heteropentanuclear Oxalato-Bridged nd–4f (n=4, 5) Metal Complexes with NO Ligand: Synthesis, Crystal Structures, Aqueous Stability and Antiproliferative Activity
Source: Chemistry. 2015 Aug 10;21(39):13703–13. doi: 10.1002/chem.201502026 (PMC4583781; doi:10.1002/chem.201502026)
Supplement: Supplementary file 1 — miscellaneous_information [file chem0021-13703-sd1.pdf]

CHEMISTRY
A European Journal

Supporting Information

Heteropentanuclear Oxalato-Bridged *n*d–4f ($n=4, 5$) Metal Complexes with NO Ligand: Synthesis, Crystal Structures, Aqueous Stability and Antiproliferative Activity

Paul-Steffen Kuhn,[a] Laura Cremer,[a] Anatolie Gavriluta,[b] Katarina K. Jovanović,[c] Lana Filipović,[c] Alfred A. Hummer,[d] Gabriel E. Büchel,[a, g] Biljana P. Dojčinović,[f] Samuel M. Meier,[e] Annette Rompel,[d] Siniša Radulović,[c] Jean Bernard Tommasino,[b] Dominique Luneau,*[b] and Vladimir B. Arion*[a]

chem_201502026_sm_miscellaneous_information.pdf

Table of Contents

Figure S1. Phosphorescence emission spectrum of complex **4**.

Figure S2. Structural formulas of the model compounds.

Figure S3. UV–vis spectra of **5** (0.35 mM) in aqueous solution measured directly after dissolution (black dashed trace) and 96 h thereafter (red trace).

Figure S4. UV–vis spectrum of **9** (0.34 mM) in aqueous solution measured directly after dissolution (black line) and 96 h thereafter (red line).

Figure S5. Concentration-effect curves of complexes **1**–**9**.

Table S1. Unit cell parameters for **3** and **4**.

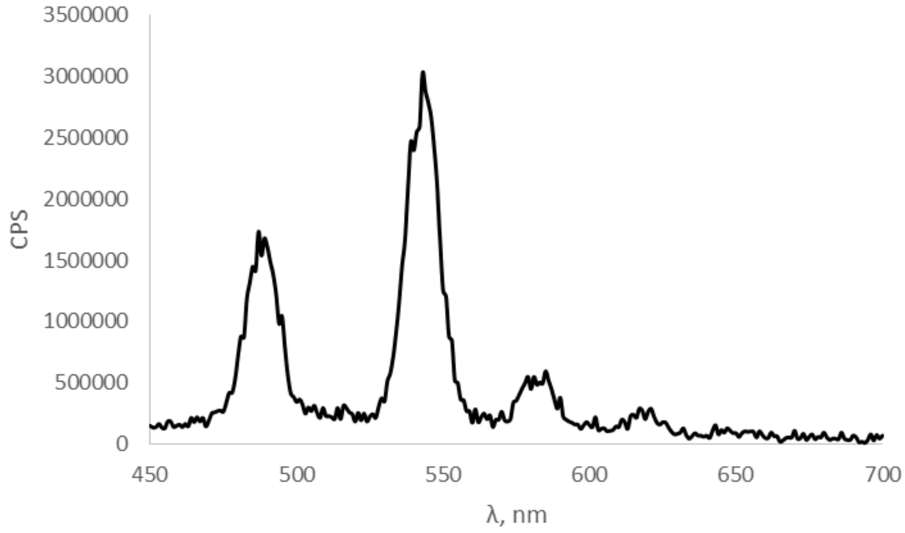

Figure S1. Phosphorescence emission spectrum (λ_{ex} = 365 nm) of complex **4**.

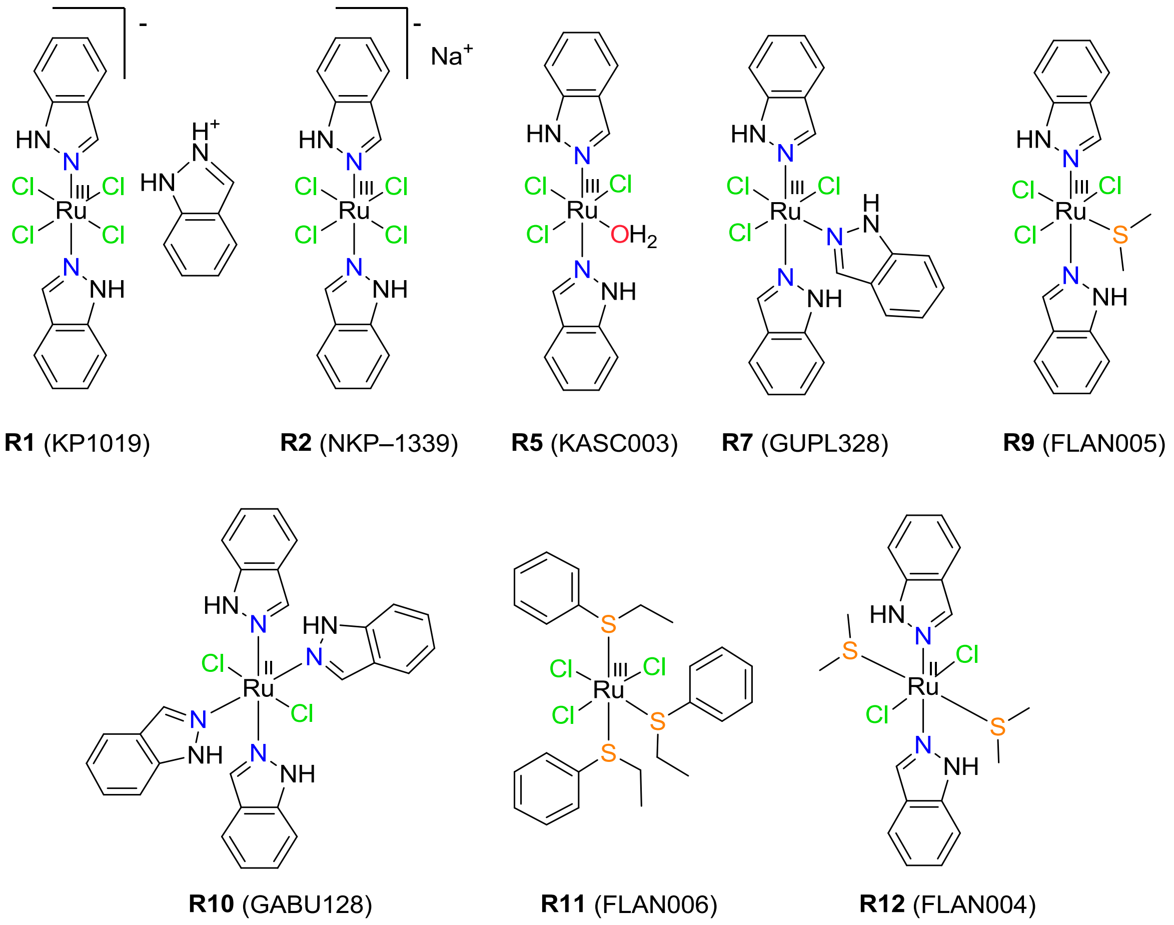

Figure S2. Structural formulas of the model compounds.

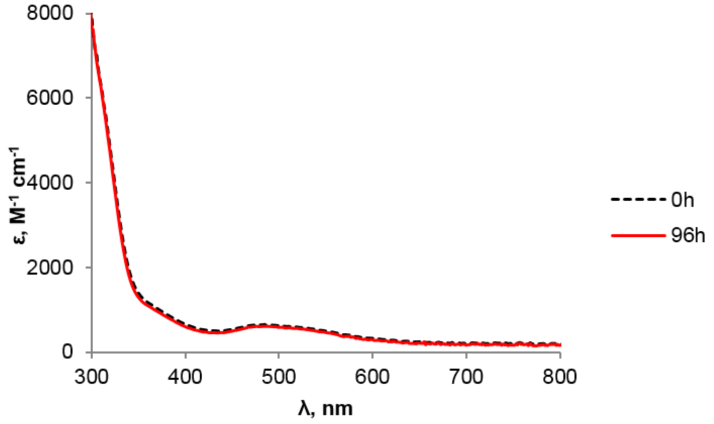

Figure S3. UV–vis spectra of **5** (0.35 mM) in aqueous solution measured directly after dissolution (black dashed trace) and 96 h thereafter (red trace).

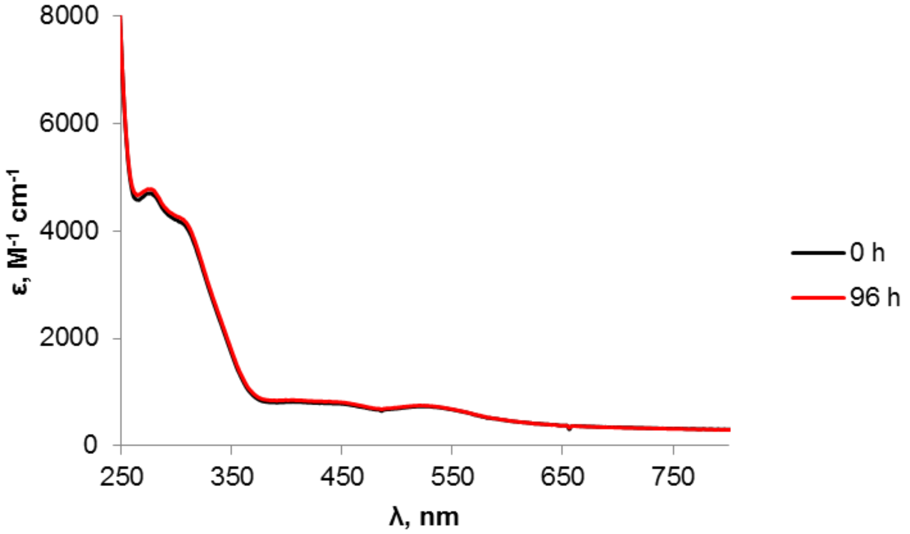

Figure S4. UV–vis spectrum of **9** (0.34 mM) in aqueous solution measured directly after dissolution (black line) and 96 h thereafter (red line).

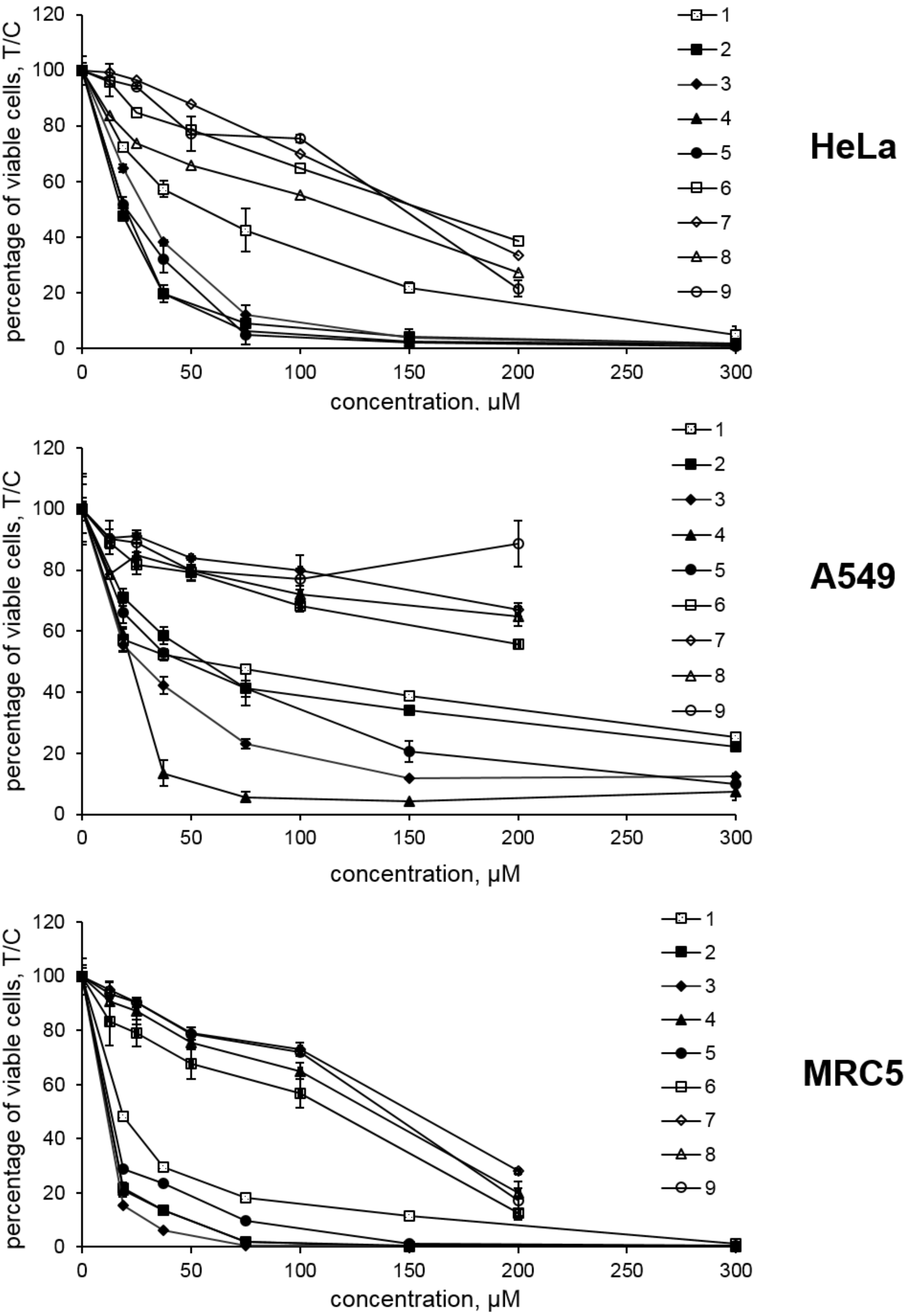

Figure S5. Concentration-effect curves of complexes **1**–**9** in the two human cancer cell lines HeLa and A549 and in the noncancerous cell line MRC-5, obtained by the MTT assay (48 h exposure).

Table S1. Crystal parameters for **3**, **4** and **4´**.

Compound	**3**	**4**	**4´**
Space group	*Cc*	*Cc*	$P\text{-}42_1c$
α, Å	17.9318	18.0098	18.4046
b, Å	27.1662	27.233	18.4046
c, Å	26.8678	26.9135	18.4366
α, °			
β, °	92.721	92.529	
γ, °			
